# Epilepsy and Issues Related to Reproductive Health

**DOI:** 10.7759/cureus.48201

**Published:** 2023-11-03

**Authors:** Muskan Khan, Nandkishor J Bankar, Gulshan R Bandre, Anupama V Dhobale, Pranita A Bawaskar

**Affiliations:** 1 Clinical Embryology, School of Allied Health Sciences, Datta Meghe Institute of Higher Education and Research, Wardha, IND; 2 Microbiology, Jawaharlal Nehru Medical College, Datta Meghe Institute of Higher Education and Research, Wardha, IND; 3 Obstetrics and Gynecology, Datta Meghe Medical College, Datta Meghe Institute of Higher Education and Research, Wardha, IND

**Keywords:** epilepsy, antiepileptic drugs, reproductive abnormalities, hormones, sexual function, infertility

## Abstract

Sexual disorders are prevalent and vary in men and women with epilepsy (WWE). Infertility and reproductive abnormalities are twice as common in females with epilepsy. Antiepileptic medications and seizures can both have an impact on sexual health in people with epilepsy. Seizures can alter the release of pituitary and hypothalamic hormones, and some antiepileptic drugs (AEDs) can alter sex steroid hormones (gonadal steroids and gonad corticoids). Females with epilepsy are more susceptible to *menstrual cycle irregularity* and *polycystic ovary syndrome*. Females and males had lower reproductive rates, and the causes are likely psychological and physiological, with epilepsy and AEDs again playing a role. Sexual disorders are common in WWE and men with epilepsy and can be caused by psychological, physical, or social factors. Specialists must address the gender-based biology of epilepsy and the impact of AEDs on sexual well-being to offer the best treatment possible for patients with epilepsy, particularly women of sexual maturity.

## Introduction and background

Epilepsy is a prevalent neurological disease that affects one in 100 people [1]. Approximately 3.4 million people in the United States have epilepsy, and up to 30% of people with epilepsy also have significant mental health problems, such as severe schizophrenia, depression, or bipolar disorder [2,3]. Furthermore, women with epilepsy (WWE) may have metabolic and reproductive health issues [4,5]. People with epilepsy constantly worry about having a seizure, having to take medications every day for years, and the economic and social difficulties that come with this undiagnosed condition [6,7]. Epilepsy has a broad spectrum of physiological consequences due to antiepileptic pharmaceutical therapy [8]. Variations in sex steroid hormones (gonadal steroids and gonad corticoids) throughout pubescence, menarche, and menstrual have been associated with the onset and progression of women's seizure disorders [2]. Some antiepileptic drugs (AEDs) lower sex steroid hormones and may affect the effectiveness of contraceptive steroids, have lower birth rates, and are more susceptible to developing infertility conditions that include disruption of the hypothalamic-pituitary-adrenal axis and anovulation with polycystic ovary-related disorders [9,10]. The impact of seizures and AEDs is considered in the best therapeutic care. When the type of seizures and general health are addressed, AEDs are improved [11]. AEDs lower the concentrations of bioavailable sex steroid hormones, affecting menstrual period control and *contraceptive effectiveness*. These medications can suppress natural *sex steroid hormones*, which can cause reproductive disorders [12]. Most of the fresh information from various research initiatives, particularly those contributing to the future pregnancy registry, can benefit doctors and professionals caring for women with epilepsy [13,14]. Concerns about sexual health hazards for WWE were recently explored in an *American Academy of Neurology practice guideline* that explains the care difficulties of females with epilepsy [2,9]. Also in pregnancies, the pharmacokinetics of the AEDs alter and may increase the frequency of seizures [15,16]. Comprehensive treatment for females with epilepsy controls seizures and protects general long-term wellness [17]. This study summarizes how epilepsy affects reproductive health.

## Review

Epilepsy is a neurological disorder characterized by unprovoked seizures, which can significantly affect the quality of life of individuals with epilepsy and can dramatically affect reproductive health issues, including fertility, contraception, pregnancy, and childbirth. This is because the hormonal shifts that take place throughout these phases have a direct bearing on the frequency of seizures and the efficacy of medicines. Achieving and sustaining a healthy pregnancy can be difficult for WWE since several anti-seizure drugs raise the risk of birth abnormalities. Furthermore, the physical strain and stress of labor might cause seizures in certain epileptics. Working closely with their healthcare professionals is essential for people with epilepsy to manage their illness and make plans for any potential difficulties related to reproductive health [3,4]. To provide people with epilepsy with the proper treatment and support, it is crucial to comprehend the intricate and multifaceted interaction that exists between reproductive health concerns and epilepsy. An irregular menstrual cycle, problems conceiving, and hormone abnormalities are just a few of the ways that epilepsy can impact reproductive health. Since epileptic seizures might pose a risk to a pregnant woman, it is important to effectively control the condition before becoming pregnant [7,11,18].

**Figure 1:**
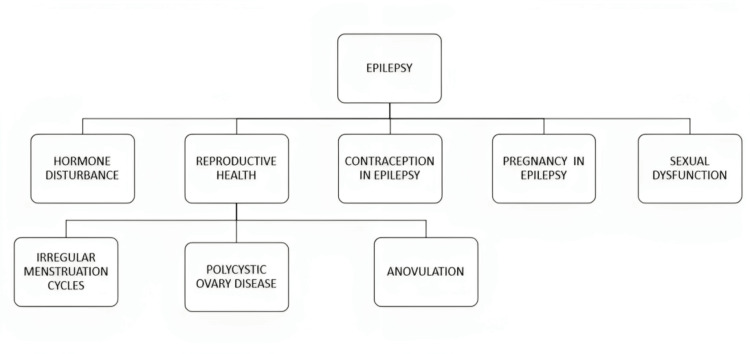
Issues of reproductive health associated with epilepsy. Sources: [2,4,7-9].

Epilepsy contributes significantly to the global burden of diseases, affecting approximately 50 million individuals worldwide [18]. At any particular time, the expected percentage of the average community with active epilepsy (continuous episodes or the need for medication) is between 4 and 10 per 1,000 individuals [19]. Each year, an estimated five million people worldwide are affected by epilepsy. Epilepsy is expected to affect 49 people out of 100,000 in high-income countries annually. In middle- and low-income nations, this number can reach 139 per 100,000 people [20]. This is most likely due to the increased risk of endemic diseases such as neurocysticercosis or malaria, the increased frequency of birth-related injuries, road traffic accidents, variations in medical facilities, the availability of preventive healthcare programs, and available treatment. Almost 80% of people with epilepsy reside in middle- and low-income nations [21]. The issue of reproductive health and epilepsy is depicted in Figure 1 [2,4,7-9].

Hormone disturbances

Epilepsy and seizures affect the levels of pituitary and hypothalamic hormones [22], and some AEDs change the amount of sex steroids generated by the adrenal glands and ovaries [23-25]. The production of anterior pituitary gonadotropin is controlled by the hypothalamus through the secretion of gonadotropin-releasing hormone (GnRH) [26]. *GnRH* is released in bursts to stimulate pituitary gonadotropin production, such as follicle-stimulating hormone (FSH) and luteinizing hormone (LH) [27]. FSH and LH stimulate the generation and release of sex steroids from the gonads, providing negative and positive feedback to the pituitary gland and the hypothalamus [28-30].

Seizures can disrupt the cortical regulation of hypothalamic hormone production, resulting in hypothalamic-pituitary axis dysfunction [8]. An irregular pituitary reaction to GnRH, a change in the pulsatile release of abnormal pituitary LH, elevated pituitary LH concentrations, and elevated prolactin are examples of endocrine abnormalities in WWE [25]. A variety of factors could cause these endocrine diseases. Ictal discharges can cause direct and episodic changes in the hypothalamic-pituitary-adrenal axis, and epileptogenic damage can cause prolonged axis dysfunction [29]. AEDs can alter the function of the hypothalamic-pituitary-adrenal axis by changing the regulation of feedback by gonadal steroid sex hormones and directly influencing the input to the axis. Endocrine disorders may arise from these modifications in the hypothalamic-pituitary-adrenal axis, which may in turn cause alterations in hormone levels. AEDs can also exacerbate these problems by disrupting the gonadal steroid sex hormones' modulation of feedback. To treat any potential endocrine issues, WWE must constantly check their hormone levels and confer with their healthcare specialists [30].

Prolactin is also raised intracranially in certain males and females with epilepsy. Pituitary prolactin levels increase more than twice as much after grand mal seizures, the most difficult focal seizures, and simple focal seizures involving brain areas, but not after a non-epileptic seizure. The climb begins within five minutes, rises after 15 minutes, and lasts one hour before returning to baseline levels. This increase in prolactin levels is thought to be caused by stress and hormonal imbalance during seizures. Increased prolactin levels may have a variety of effects on the body, including suppressing ovaries in females and causing sexual dysfunction in males and females. Understanding the fluctuations in the prolactin levels during seizures can provide valuable insights into the basic mechanisms of epilepsy and may lead to the development of new treatment approaches [31].

AEDs affect the hypothalamus-pituitary-adrenal axis by directly belonging to the cortical inputs to the axis and changes in response control provided by gonadal steroid sex hormones. AEDs influence neurochemicals that regulate the hypothalamic-pituitary-adrenal axis, such as *gamma-aminobutyric acid*, endogenous opioids, serotonin, and glutamate [32]. Certain AEDs also affect steroid hormones that change brain and pituitary feedback. Steroid metabolism and steroid hormone binding are improved by AEDs that stimulate cytochrome P450 proteins [33]. Thus, lowering the hormone levels accessible to the central nervous system (CNS). AEDs that reduce steroid hormone concentrations include oxcarbazepine, carbamazepine, phenytoin, topiramate, and phenobarbital [34]. Furthermore, AEDs inhibit this enzyme pathway and increase the levels of steroid hormones. A new study has found that adrenal and gonadal hormones in women taking lamotrigine or gabapentin alone showed zero difference in the non-epileptic control [35,36]. Doctors must also be on the lookout for seizures, AED-related reproductive hormonal abnormalities, and ovarian dysfunction. Changes in the duration or regularity of the menstrual period are a good indicator of anovulatory periods. Hirsutism, overweight, or pimples are all symptoms of increased androgens and androgen hypersensitivity [37]. Hyperandrogenism can be accompanied by lipid problems and glucose intolerance, which can have severe long-term health consequences [38].

Reproductive health and epilepsy

Reduced reproductive rates can be attributed to a variety of factors. According to a study, people with epilepsy are less likely to get married and have kids [39]. The risks of pregnancy and fetal birth associated with motherly epilepsy were not well understood, according to a recent survey of medical professionals who would likely interact with WWE. Many doctors also disagreed with an woman with epilepsy getting pregnant because they were worried about possible harm to the unborn child as well as the mother. Pregnancy presents particular difficulties for those with epilepsy, a neurological condition marked by recurring seizures. The necessity for thorough education and training in this area is highlighted by the ignorance of medical professionals regarding the unique hazards and management techniques for WWE during pregnancy. However, it's crucial to remember that each woman's circumstances are different, and choices about getting pregnant should be taken on an individual basis while considering the person's general health and seizure control [40,41].

Irregular Menstrual Cycles, Polycystic Ovary, and Anovulation

WWE are much more susceptible to abnormal menstrual cycle durations, such as minimal periods <23 days and excessively long cycles >35 days. According to a recent study, 28.8% of WWE had highly irregular periods. WWE have around one-third of their anovulatory menstrual cycles, compared to approximately 12% of women without epilepsy [42]. Females with initial generalized epilepsy are more likely to have anovulation periods than females with related localized epilepsy. AEDs were shown to be significantly associated with anovulation periods, but not gabapentin, phenobarbital, carbamazepine, phenytoin, and lamotrigine. Females with primary generalized epilepsy who were on AEDs were the most vulnerable [43]. Fifty-five percent of the menstrual cycles in this group were anovulatory. Ovulatory failure induced by epilepsy, and some AEDs may be the result of hormonal abnormalities and ovarian dysfunction [34]. The fact that the release of pituitary LH in WWE changes freely and in response to GnRH shows that the hypothalamic-pituitary axis is dysfunctional [44]. Women who used AEDs that activate cytochrome P450 proteins experienced significant decreases in blood, testosterone, and estradiol levels, with higher levels of sex steroid globulin [45]. Women who used AEDs with the cytochrome P450 protein inhibitor had considerably higher adrenal and gonadal androgens [46]. WWE who use lamotrigine or gabapentin, two AEDs that do not affect cytochrome P450 proteins, have a hormonal balance that is not different from non-epileptic controls [47,48].

Obesity, polycystic ovaries, acne, hirsutism, elevated FSH: The LH ratio, increased androgens, impaired insulin sensitivity, and prolonged anovulation are all symptoms of polycystic ovarian syndrome [49]. This syndrome has been associated with infertility, in addition to a higher risk of heart disease, dyslipidemia, diabetes, impaired insulin sensitivity, endometrial carcinoma, and perhaps breast carcinoma throughout the breast throughout a lifetime [36]. Although most women with polycystic ovary syndrome (PCOS) who have epilepsy have not been evaluated for other elements of this syndrome, there is some evidence that none of the diagnostic criteria is met. Anovulation frequently results in erratic or nonexistent menstrual periods. There is a considerable reduction in the likelihood of pregnancy without regular ovulation. Amenorrhea (the lack of menstruation) or irregular bleeding signs may result. The most typical cause of anovulation is PCOS. Its defining features are multiple tiny ovarian cysts, hormonal abnormalities, and frequent insulin resistance. Anovulation may also be linked to other health hazards, including a heightened chance of endometrial hyperplasia (uterine lining expansion) and, over time, a marginally increased risk of endometrial cancer. The illness is caused by antiepileptic medications [50]. Maintaining an appropriate weight through a balanced diet and regular exercise helps control ovulation, especially when weight issues are an issue. Much stress might mess with your hormone balance. Yoga, meditation, and relaxation exercises are examples of techniques that may be helpful. In many instances of anovulation, ovulation can be stimulated by fertility medications like clomiphene citrate or letrozole. In a small group of women with symptoms of polycystic ovary disease such as *hyperandrogenism*, *polycystic ovary*, and *dyslipidemia* manifested as *high-density lipoprotein* and an elevated *low-density lipoprotein* cholesterol level [40]. AEDs were replaced with lamotrigine [48]. The switch from AEDs to Lamictal resulted in reverse hyperandrogenism, normalization of ovarian morphology, and lipid profile, including increased HDL levels [49,51]. Table 1 shows conditions and their potential relationship with epilepsy [42,43,47,48].

**Table 1:** Condition and their potential relationship with epilepsy. Sources:  [42,43,47,48]. PCOS, polycystic ovary syndrome

Condition or term	Definition/description	Relationship with epilepsy
Irregular menstrual cycles	Menstrual cycles that differ from the standard 28-day cycle, including changes in cycle duration and abnormal bleeding patterns.	Some women with epilepsy may have irregular menstrual periods due to hormonal changes, stress, or drugs. Menstrual irregularity can be affected by epilepsy, which can disturb the endocrine system.
PCOS	A hormonal condition that can result in larger ovaries with tiny cysts on the outside margins. Period irregularities, excessive hair growth, and hormone abnormalities are common symptoms.	Perhaps because of similar hormone imbalances, PCOS is more common in women with epilepsy. The symptoms of PCOS may worsen when taking some antiepileptic drugs.
Anovulation	The lack of ovulation stops the ovaries from releasing a mature egg. Menstrual cycles may become irregular or nonexistent as a result.	Anovulation is a possibility for certain women with epilepsy, potentially as a result of hormonal imbalances brought on by the condition or its treatment. The hypothalamic-pituitary-ovarian axis can be upset by epileptic episodes, which might impact ovulation.

Contraception and epilepsy

Contraception is essential for people with epilepsy who wish to avoid unwanted pregnancy. However, the choice of contraception can be challenging, as some contraceptive methods can interact with AEDs or have reduced efficacy in individuals with epilepsy. Individuals with epilepsy need to consult with their healthcare provider to find a suitable contraception method that does not interfere with their AEDs and provides effective protection against unplanned pregnancies [52]. Hormonal contraceptives such as the pill, patch, or hormonal intrauterine devices (IUDs) may require adjustments in dosage or type of AEDs to ensure maximum effectiveness. Nonhormonal options like barrier methods or copper IUDs can be considered as well, depending on the individual's specific needs and medical history [53,54].

AEDs can interact with hormonal contraceptives, such as combined oral contraceptives, reducing their efficacy and increasing the risk of breakthrough seizures [12]. A study by Halane et al. reported that enzyme-inducing AEDs, such as carbamazepine and phenytoin, can reduce the efficacy of hormonal contraceptives, leading to unplanned pregnancies in WWE [53]. Therefore, WWE who use hormonal contraceptives should be advised to use additional contraception, such as condoms, or consider alternative contraceptive methods, such as copper IUDs or progestin-only contraceptives, which are not affected by AEDs. In addition to hormonal contraceptives, contraceptive barriers such as condoms, diaphragms, and cervical caps are safe and effective for patients with epilepsy and do not interact with AEDs [55,56]. However, patients with epilepsy may have increased seizure activity during sexual activity, affecting the ability to use contraceptive barriers. Therefore, physicians should discuss the risks and benefits of various contraceptive methods with epilepsy patients and consider their seizure control when making contraceptive recommendations. Similar to healthy women, women with epilepsy (WWE) use a variety of contraceptive techniques, including IUDs or combinations of these, hormonal contraception (HC) such as progestin-only tablets, barrier methods, subdermal implants, intramuscular injections, hormone-releasing skin patches, and vaginal rings. Additionally, the majority of WWE also use AEDs. Numerous medications interact with HC, which might result in contraceptive letdown or poor seizure management [57].

Pregnancy and epilepsy

Pregnancy can be challenging for WWE, as they can experience changes in the frequency of seizures, increased complications, and the need to manage their epilepsy medication during pregnancy. However, with proper care and effort, most WWE can successfully conceive and deliver a healthy baby. Changes in the frequency of seizures during pregnancy are unpredictable, with some women experiencing an increase and others experiencing a decrease. During pregnancy, 33% of WWE experienced increased seizures, 24% experienced a reduction, and 43% reported no change in the frequency of seizures [58]. Therefore, it is essential to monitor seizure activity during pregnancy and adjust epilepsy drugs to optimize seizure control. The use of AEDs during pregnancy also affects the development of the fetus and increases the risk of congenital malformations [59]. The risk of congenital malformation varies depending on the type and dose of AEDs used, and some AEDs are associated with higher risk than others. For example, valproate has been associated with an increased risk of neural tube defects and other congenital malformations. Therefore, clinicians should carefully consider the risks and benefits of AED treatment during pregnancy and consider alternative treatment options or reduce the dose of AEDs as needed to minimize the risk of congenital malformations. AED exposure to fetuses should be minimal for best seizure control. Major congenital malformations (MCM), dysmorphic syndromes, intrauterine growth retardation, and abnormalities in neurocognitive development may all be linked to prenatal exposure to AEDs. AED pharmacokinetics are altered by physiological changes during pregnancy, which may lead to decreased levels and worsening seizures in WWE, although therapeutic drug monitoring and adjusting the dose of AEDs during pregnancy and postpartum can prevent this [60].

Sexual disorder

Another cause of low birth rates and a clinical problem is epilepsy-related sexual dysfunction. Men with epilepsy and WWE have a far higher incidence of sexual difficulties [61]. When we consider people with other chronic neurologic conditions, the disorder presents mainly as a decrease in sexual drive and potency. Thirty percent to 66% of men with epilepsy and 14% to 50% of WWE are affected by a sexual disorder [62]. Males with epilepsy have sexual concerns such as lack of spontaneous nocturnal erections, erectile dysfunction, and anorgasmia [40,50]. Studies suggest that more than one-third of WWE have vaginismus, lack of vaginal lubrication, and dyspareunia when they have normal sexual desire and experience [40,63]. The presence of sexual disorders in individuals with epilepsy is likely to be complex. The social development of some epileptic sufferers is hampered. Seizures can cause low self-esteem, contributing to perceptions of sexual undesirability [64]. Epileptic discharge in areas of the brain that mediate sexual conduct may potentially play a role in sexual disorder [65,66]. The sexual disorder is associated with changes in pituitary gonadotropins. Elevated prolactin, progesterone, and testosterone levels and low estrogen level are related to sexual disorders in WWE [26]. Impotent men with epilepsy have higher amounts of estradiol. Some antiepileptic medications cause sexual diseases directly or indirectly through hormone changes that stimulate sexual activity [30].

Evaluation of sexual dysfunction

Patients with epilepsy may experience sexual dysfunction, a complex problem resulting from both neurological abnormalities and the side effects of AEDs. Sexual dysfunction may result from seizures and the impact of epilepsy on hormone balance. Furthermore, libido, arousal, and orgasm may be negatively impacted by the adverse effects of several antiepileptic medications. Anxiety, sadness, and body image issues are psychological variables that might worsen the issue. Open communication between spouses and a multidisciplinary approach comprising psychologists, gynecologists, and neurologists are crucial to addressing this issue [1,6,67]. An assessment of the patient's needs, the epileptic condition and any comorbidities, and the medications available for controlling epilepsy are all necessary for determining the best course of action for individuals with sexual dysfunction who are epileptic. Various strategies, including behavioral techniques to enhance sexual performance, customization of the extensive range of available antiepileptics for individual patients, dosage reduction of current medications, waiting for tolerance to develop, adjuvant treatment for sexual dysfunction, delaying drug administration until after sex, and targeted treatment for sexual dysfunction itself, may contribute to the management of sexual dysfunction in patients with epilepsy. They are providing various patients with access to a wide range of antiepileptic medications [10,44,63].

AED-related sexual dysfunction is treated with several adjuvant medications, including buspirone, cyproheptadine, yohimbine, buspirone, neostigmine, amantadine, mianserin, and dexamphetamine. Testosterone and aromatase inhibitors have been used in the experimental environment to treat sexual dysfunction in males taking AEDs. For patients with epilepsy, patient education and follow-up sessions are crucial to achieving the best possible results from pharmacologic therapy for sexual dysfunction. There are currently no authorized pharmaceutical therapies for female orgasmic dysfunction and hypoactive sexual drive. Nonetheless, vaginal lubricants or estrogen replacement treatments treat female sexual arousal issues [36,44]. The most invasive forms of reproductive treatment include in vitro fertilization and intracytoplasmic sperm injection. To stimulate ovulation, the *human chorionic gonadotropin* and the FSH are used, after which the ova are recovered by transvaginal aspiration and fertilized in vitro. A single sperm is inserted into the oocyte during intracytoplasmic sperm injection [68].

## Conclusions

Women are especially concerned about epilepsy throughout their reproductive age. Infertility rates are higher due to psychological stress faced by people with epilepsy and impaired physiological processes that maintain reproductive health. Finally, the physician must examine the physiological implications of antiepileptic and seizure medications. The goal is to provide the WWE with free-from-seizure living, excellent overall health, and improved health. The current plan is achievable if the healthcare practitioner is familiar with the biology of gender-based epilepsy. The limitation is these treatments can not be affordable to every woman in the resource-limiting setting.
